# Editorial: The Role of Nutrition in the Management of Liver and Associated Diseases

**DOI:** 10.3389/fnut.2022.919057

**Published:** 2022-06-10

**Authors:** Naga Betrapally

**Affiliations:** Laboratory of Integrative Cancer Immunology (LICI), Center for Cancer Research (CCR), National Cancer Institute (NCI), Rockville, MD, United States

**Keywords:** microbiome, nutrition, short chain fatty acids (SCFA), ammonia, NAFLD

The liver is a vital organ that plays an important role in nutrient capture, detoxification, and metabolizing drugs in the human body. Management of the health of the liver is a bi-directional relationship between the liver and the gut microbiome that is carried out *via* multiple functions (1). A malfunctioning liver is associated with various health concerns such as obesity leading to non-alcoholic fatty liver disease (NAFLD). NAFLD is a metabolic stress-related liver injury involving metabolic dysfunction and genetic susceptibility. It has become the most common cause of chronic liver disease worldwide, affecting 25% of the global population to some degree (2).

This recent Research Topic, “*The role of Nutrition in the Management of Liver and Associated Diseases*,” represents a Research Topic of five articles, including three original research articles that focus on the impact of the Gut Microbiome in the regulation of NAFLD both in adolescents and adults, the impact of dietary interventions, nutrient uptake, and effects of ammonia on NAFLD. A common theme across the Research Topic is the assessment of factors contributing to NAFLD and its prevention. This is an important topic for investigation in understanding how sarcopenic obesity, dietary protein, dietary interventions, and the role of microbiome impact the overall outcome for NAFLD.

Dong et al. examine the role of the microbiome in clinical response by examining weight loss and change in hepatic steatosis on a calorie-restricted diet. They examined 80 subjects in the age group between 20 and 75 years with a BMI of at least 27 kg/m^2^ on a macronutrient diet by varying protein and carbohydrate intake for 2 weeks and transitioning to calorie restricted diet with the same macronutrient profile for 14 weeks. The subjects demonstrated a significant reduction in weight, BMI, cholesterol, low density lipoprotein, and trigylcerides along with significant baseline microbiome differences between subjects who had at least 5% weight loss with an overall improvement in hepatic steatosis. A dysbiotic gut microbiota alters the gut-liver homeostasis by disrupting the gut barrier, portal transport of bacterial endotoxin to the liver, altered bile acid profiles, and decreased concentrations of short-chain fatty acids (3). A distinct microbiome signature during neonatal and infant periods are correlated with breastfeeding, formula ingredients, mode of delivery, maternal gestational weight gain, and gender in healthy children. A decreased presence of specific bacteria, such as *Faecalibacterium prausnitzii*, bacteria of genus *Oscillospira*, have been shown to be associated with NAFLD. A disruption of the gut barrier by variation in lifestyle induced dietary composition, duration, and feeding patterns and is enhanced by antibiotic use during childhood. Tokuhara discusses the mechanisms and maternal influences along with conventional and gut microbiota targeted preventatives and treatments for NAFLD.

Nutrition plays an important role in management of NAFLD, with interventions having the ability to halt or reverse NAFLD progression (4). Shi et al. discuss the impact of dietary protein on lipid metabolism and oxidative phosphorylation in rat liver based on meat source. The authors investigate the effect of different dietary meat proteins on gene expression and propose the relationship of body weight, blood lipid, and amino acids with hepatic lipid metabolism, amino acid metabolism, and pancreatic islet signaling pathway. They highlight the effects of cholesterol by studying genes related to liver cholesterol synthesis in rats fed with different types of meat protein and energy metabolism. The triglyceride levels in liver were lower in protein diet groups and the degradation and metabolism of triglyceride were promoted, suggesting an association to meat diet and human health.

The presence of NAFLD in non-obese patients remains unclear. A reduction in the existing muscle mass is seen in such patients. Chronic liver diseases can lead to hyperammonemia which contributes to muscle depletion through diverse mechanisms such as increasing the expression of myostatin or impairing bioenergetics in the muscle (5). Wang et al., designed a study to identify the effects of high fat diet and tested an ammonia lowering drug for efficacy in the treatment of steatohepatitis and sarcopenia. Their results show that ammonia lowering drugs can reduce plasma ammonia and restore muscle mass and strength in mice with a high fat diet. This work suggests that lowering ammonia could be a promising therapeutic strategy in NAFLD.

A thorough review by Houttu et al. provides insights into the interventions strategy to establish the effect of different dietary modifications on lipid content, liver fibrosis, and function in patients with NAFLD. Their review focuses on identifying dietary interventions without any exercise focusing on body weight, glucose metabolism, and plasma lipid profiles.

In summary, this volume brings togethe a number of papers that provide insights into calorie deficit diets and the role of the microbiome in predicting and managing NAFLD both in adults and adolescents, as well as possible treatments such as ammonia reduction in NAFLD. This reading will certainly provide new information for clinicians and researchers in the field of nutrition and liver diseases, especially NAFLD.

## Author Contributions

The author confirms being the sole contributor of this work and has approved it for publication.

## Conflict of Interest

The author declares that the research was conducted in the absence of any commercial or financial relationships that could be construed as a potential conflict of interest.

## Publisher's Note

All claims expressed in this article are solely those of the authors and do not necessarily represent those of their affiliated organizations, or those of the publisher, the editors and the reviewers. Any product that may be evaluated in this article, or claim that may be made by its manufacturer, is not guaranteed or endorsed by the publisher.
